# The Synergistic Production Effect of Water and Nitrogen on Winter Wheat in Southern Xinjiang

**DOI:** 10.3390/plants13101391

**Published:** 2024-05-17

**Authors:** Qingyuan Lei, Wanghai Tao, Shudong Lin, Lijun Su, Mingjiang Deng, Quanjiu Wang, Fan Yang, Tao Zhu, Liang Ma

**Affiliations:** 1 State Key Laboratory of Eco-Hydraulic in Northwest Arid Region, Xi’an University of Technology, Xi’an 710048, China; leiqy9527@163.com (Q.L.); xautsoilwater@163.com (W.T.); shudong_lin@163.com (S.L.); sljun11@163.com (L.S.); wquanjiu@163.com (Q.W.); yf22283435311013@163.com (F.Y.); 2 College of Water Conservancy and Civil Engineering, Xinjiang Agricultural University, Urumqi 830052, China; 18692730396@163.com

**Keywords:** dry matter, photosynthetic characteristics, water-and-nitrogen-use efficiency, winter wheat, yield

## Abstract

Water and nitrogen management are crucial for food security and the efficient use of water and fertilizer, especially in arid regions. Three irrigation levels, namely, 80% crop water requirement (*ET_C_*) (W1), 100% *ET_C_* (W2), and 120% *ET_C_* (W3), and three nitrogen application levels, namely, 0 kg/ha (N1), 207 kg/ha (N2), and 276 kg/ha (N3), were used as the experimental treatments, and a control group, denoted as CK, was created. The results show that the maximum height achieved was 82.16 cm under W3N3. There was a single-peak variation trend throughout the growth stages of *SPAD*. It peaked at 58.44 under W3N3 and then at 27.9 under W2N2. The net photosynthetic and transpiration rates displayed bimodal peaks and the phenomenon of a “photosynthetic midday depression”. And the prominent peaks in leaf water use efficiency occurred at 14:00 and 18:00, alongside noteworthy enhancements observed under the W3 treatment. Water and nitrogen and their interactions significantly affected the dry matter (*DM*) of winter wheat, with the spike accounting for the highest percentage. The W2N2 treatment demonstrated superior effectiveness in enhancing winter wheat water use efficiency, offering the potential to decrease irrigation requirements by 20% and nitrogen application by 25%. Moreover, the maximum *PFPN* attained under W2N2 reached 60.13, representing a noteworthy 35.25% increase compared to the control group (CK), but the *HI* of the W2N2 treatment only reached 0.56. The highest *HI* was achieved with W3N2 (0.73), and the nitrogen application of 207 kg/ha was more conducive to obtaining a higher *HI*. The highest yield was achieved under W3N3 (13.599 t/ha), followed by W2N2 (12.447 t/ha), and the spike proportion exceeded 60% with W2N2, and its production cost and economic benefit ratio of under 0.31 were superior to those for other treatments. Multiple regression analysis revealed that the maximum yield reached 12.944 t/ha with an irrigation amount of 3420.1 m^3^/ha and a nitrogen application of 251.92 kg/ha. Overall, our study suggests using an optimal water–nitrogen combination, specifically an irrigation level of 2829 m^3^/ha and a nitrogen application rate of 207 kg/ha, leading to increased winter wheat yields and economic benefits. These research results provide a pragmatic technique for improving winter wheat production in southern Xinjiang.

## 1. Introduction

Winter wheat (*Triticum aestivum*) is one of the three major food crops worldwide, and its stable and efficient production is crucial to ensure food security [1]. Northwest China accounts for 35.9% of the total national wheat-producing area. In comparison, the water resources in the same region only account for 5.7% of the national total [2]. Xinjiang is a substantial grain production base in China, with wheat serving as its foremost crop [3]. Southern Xinjiang is a typical arid region [4], characterized by abundant light and heat and significant temperature differences between day and night. At the same time, local water shortages and inefficient water and fertilizer application are not conducive to the long-term development of agriculture [5]. Therefore, addressing water resource shortages and other issues threatening agricultural production in arid and semi-arid areas is essential [6] to facilitate higher crop yields. Efficient water and nitrogen regulation is an integral component of increasing farmland yields [7]. An inevitable trend of water-saving technology in Xinjiang Oasis agriculture is the implementation of irrigation and fertilization based on a water–nitrogen coupling mechanism to facilitate integrated water and fertilizer management [8]. Optimizing water and nitrogen management strategies has become imperative for sustainable agricultural production.

Compared to previous studies focusing solely on either water or nitrogen management measures, the synergistic effect of coupling water and nitrogen is more significant than the impact of irrigation or fertilization alone [9], and improving water and nitrogen utilization rates is necessary to achieve a high yield from grain crops [8,10]. The water–fertilizer integration technology of drip irrigation can transport water and nutrients to the vicinity of crop roots [11], thus applying a coupling effect of water and fertilizer. This method has been widely used in the production of food crops and is the most effective means of saving water and improving water efficiency and nitrogen utilization in arid areas [12]. By regulating the application period for nitrogen fertilizer and the irrigation cycle, low-intensity and high-frequency nitrogen applications can effectively promote winter wheat growth and biomass production [13,14].

However, while optimizing growth indicators like crop height through field water and fertilizer management is essential, achieving higher yields requires more than just promoting winter wheat growth and biomass production. Functional efficiency parameters such as net photosynthetic rate (*Pn*), transpiration rate (*Tr*), chlorophyll content (*SPAD*) [15], and leaf water-use efficiency (*L_WUE_*) are also important for improving the accumulation of photosynthetic compounds that are vital for good crop yields [16]. For example [17], a field experiment revealed that fertilizer application significantly enhanced fruit quality as a result of improving fruit chemical composition and yield. Furthermore, improving the photosynthetic performance of flag leaves resulted in higher winter wheat yields, and photosynthate in the late growth stage contributed to the yield of wheat [18]. Dry matter (DM) is not only the product of photosynthesis but also the material basis of economic yield, and its cumulative mass is of great significance. Grain filling is the critical period for wheat DM and grain yield, with the final DM accumulation and yield being determined by photosynthetic performance at this growth stage [19]. However, the coupling effect between water and nitrogen has a threshold, and surpassing it has antagonistic effects [20,21,22]. The water-use efficiency (WUE) of winter wheat shows a decreasing trend with increasing irrigation levels. Moreover, in one study, the correlation between flag leaf photosynthetic parameters and WUE was higher during the middle stage of grain filling than in the early stage [23].

The study of water-use efficiency is inseparable from the study of nutrient-use efficiency. The partial productivity efficiency of nitrogen fertilizer and the harvest index (*HI*) are often used to evaluate crop yield and constitute two more valuable measures for quantifying both crop yield and associated economic benefits. The yield parameters of winter wheat that can be measured directly include the number of effective panicles per unit, the number of grains per panicle, and the 1000-grain mass. Ref. [24] found that the 1000-grain mass of wheat is also dependent on the grain-filling stage. These factors are all affected by field water and nitrogen application, impacting water- and nitrogen-use efficiency. However, in agricultural production, producers often over-irrigate and over-fertilize to achieve high yields [25], generating adverse consequences such as high production costs and low water- and nitrogen-use efficiency [26]. There is currently a lack of research on the water and nitrogen application thresholds of winter wheat production in Southern Xinjiang. Therefore, to achieve both a high yield and efficient production of wheat in this region, the effect of water and nitrogen coupling, using drip irrigation, on the growth and photosynthetic physiological parameters of winter wheat must be clarified.

Efficient management of water and nitrogen in winter wheat cultivation within the arid zones of Southern Xinjiang will positively influence crop yield and increase economic benefits. Exploring the complex relationships between water and nitrogen management and their impacts on winter wheat cultivation in arid regions, especially in Southern Xinjiang, is crucial for optimizing local water and nitrogen inputs and enhancing winter wheat productivity and economic returns. With this overarching aim in mind, this study set out to achieve several objectives: (1) comprehensively evaluate the effects of varying irrigation and nitrogen application levels on a multitude of parameters crucial for winter wheat growth and development; (2) identify the most effective combination of water and nitrogen inputs for maximizing winter wheat productivity while minimizing resource usage and environmental impacts; and (3) enhance the sustainability and economic viability of winter wheat cultivation in arid regions by optimizing water–nitrogen combinations to improve efficiency and boost yields. In summary, this study offers valuable guidance for agricultural producers aiming to enhance productivity, profitability, and sustainable resource management via optimal water and nitrogen management. Additionally, this study’s findings can offer essential empirical data that can bolster local production methods.

## 2. Materials and Methods

### 2.1. Research Area

**Figure 1:**
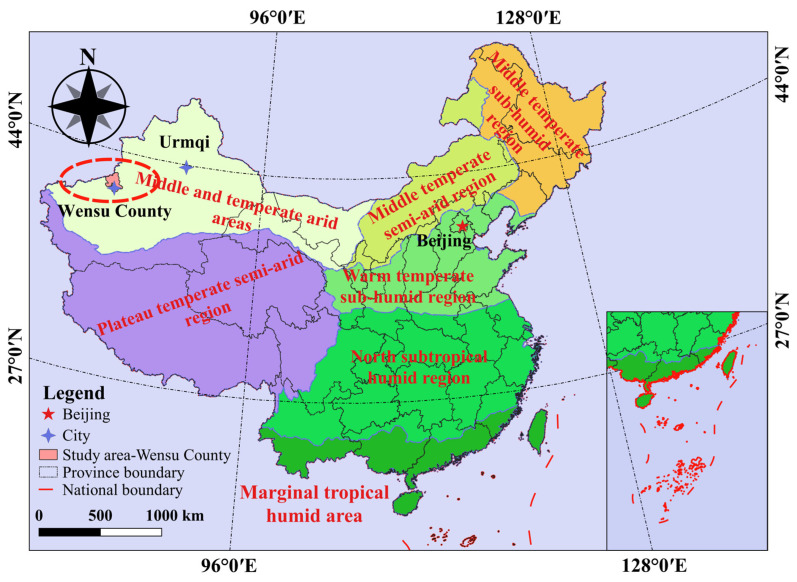
Schematic diagram of the location of the study area and the climatic region in which it is situated.

The field experiment was carried out in the characteristic forest and fruit grounds (80°14′ E, 41°16′ N, 1133 m) of Xinjiang Agricultural University, Aksu Prefecture, Xinjiang Uygur Autonomous Region, China (Figure 1), from 2017 to 2018. The study site was located on the northern margin of the Tarim Basin, which has a typical temperate continental climate. The annual total solar radiation in this area is 544.115–590.156 kJ/cm^2^, the annual duration of sunshine is 2855–2967 h, the annual amount of precipitation is 42.4–94.4 mm, the annual average temperature is 11.2 °C, and the annual effective accumulated temperature is 3950 °C, featuring extensive evaporation, a frost-free period of 205–219 days, and an underground water depth >10 m.

The soil texture in this area is loam. The soil properties and initial nutrient content obtained before winter wheat sowing are shown in Table 1.

### 2.2. Experimental Design

**Figure 2:**
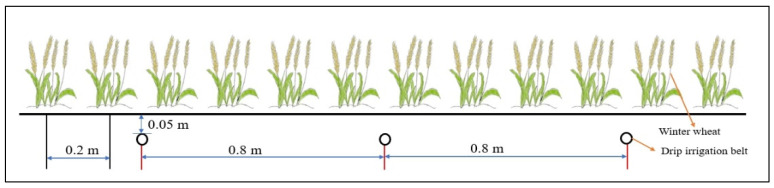
The winter wheat planting and drip irrigation belt layout in the study plots.

The variety of wheat tested was Xindong 22, with a growth period of 255 days (sown on 13 October 2017 and harvested on 25 June 2018). The seeding density was 300 kg/ha, the experimental plots measured 6.6 m^2^ (length × width × depth = 3 × 2.2 × 2 m), and the spacing between two rows of winter wheat was 0.2 m. Each plot was provided with a 0.1 m concrete boundary to prevent infiltration of external water and fertilizer. A shallow underground drip irrigation system was used; the drip irrigation belt was buried 0.05 m underground, the drip head spacing was 0.2 m, and the drip head flow was 1.8 L/h. One drip irrigation belt fed four rows of winter wheat. The layout of the plots is shown in Figure 2.

Three replicates of nine treatments were set up (Table 2). Three experimental irrigation levels were used: 80% crop water requirement (*ET_C_*) (W1), 100% *ET_C_* (W2), and 120% *ET_C_* (W3). The three experimental nitrogen fertilizer application rates used were 0 kg/ha (N1), 207 kg/ha (N2), and 276 kg/ha (N3). A plot representing the local winter wheat water and nitrogen application treatment (CK) was also set up in accordance with the recommendations of the Agricultural Technology Extension Center for the Aksu Region of Southern Xinjiang: flood irrigation was performed every 15 days after the jointing period; the irrigation quotas were 1050, 975, 900, and 600 m^3^/ha; and the pure nitrogen application rate was 207 kg/ha. Because the excessive application of nitrogen fertilizer with a water deficit is not conducive to crop growth, the low-water and high-fertilizer treatment (W1N3) was not used.

This experiment adhered to a randomized complete block design; therefore, all treatments were repeated three times. The total application amount of phosphorus pentoxide was 195 kg/ha, and the total application amount of potassium magnesium sulfate fertilizer was 105 kg/ha in each experimental plot. Fifty percent of both and fifty percent of each nitrogen treatment (N1, N2, and N3) were incorporated into the soil as basal fertilizer before seeding. The remaining fertilizer was applied using water drops in a ratio of 2:2:1 in the regreening stage, jointing stage, and heading stage, respectively. Field management measures, such as disease, pest, and weed control during the growth period, were applied according to local practices.

**Figure 3:**
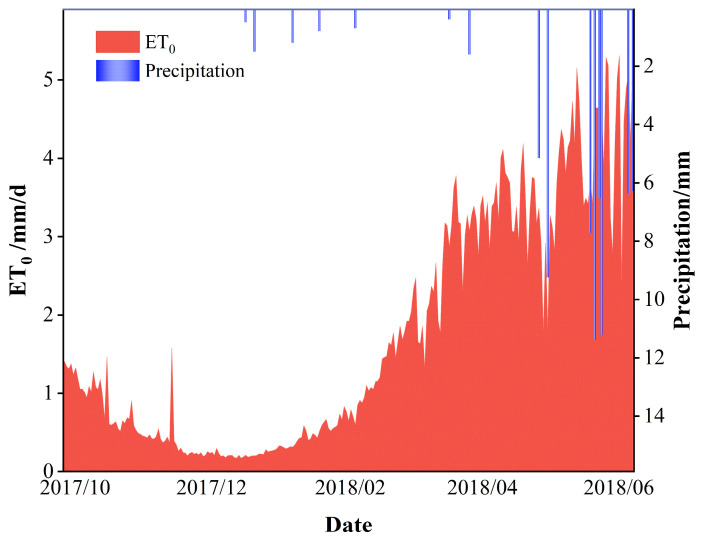
*ET*_0_ and precipitation during the winter wheat growth period.

To ensure seedling emergence, winter and spring irrigation were conducted in every experimental plot, with an irrigation amount of 900 m^3^/ha for both winter and spring, every 7 days from the jointing growth stage until the end of May. The crop water requirement (*ET_C_*) was used to calculate each irrigation quota accurately, using the following formula:

(1) $$ET_{C}=K_{C}\times ET_{0}$$

where *ET_C_* is the water requirement of crops (mm), *ET*_0_ is the reference crop transpiration rate (mm), and *Kc* is the crop coefficient. *ET*_0_ was derived from meteorological data from the prior irrigation cycle using the FAO Penman–Monteith formula (Figure 3). Subsequently, *ET_C_* could be computed by multiplying the crop coefficients (*Kc*). *Kc* was determined using the winter wheat mono-crop coefficient table recommended by FAO-56 and the associated literature (In Table 3).

The winter wheat reference evapotranspiration (*ET*_0_) and precipitation throughout the entire growth period are depicted in Figure 3:

### 2.3. Data Collection

#### 2.3.1. Meteorological Data

An automatic HOBO monitoring weather station was installed at the experimental site. The meteorological data were recorded in real time every 30 min and included meteorological parameters such as temperature, effective solar radiation, wind speed, wind direction, rainfall, relative humidity, and atmospheric pressure during the growth period.

#### 2.3.2. Photosynthetic Data

Three flag leaves were selected from each plot during the grain-filling period on a clear and windless day. The CIRAS-3 photosynthetic measurement system produced by PP Systems (USA) was used to measure the net photosynthetic rate (*Pn*), transpiration rate (*Tr*), and leaf water use efficiency (*L_WUE_*). The measurement period was from 10:00 to 18:00. Observations were made every 2 h, and average values were determined. A *SPAD*-502 chlorophyll meter produced by SONY was used to determine the relative content of chlorophyll (*SPAD*) in the flag leaves.

#### 2.3.3. Soil Moisture

A TRIME-IPH soil moisture meter was used to measure the soil moisture content for the 0–100 cm underground layer. Crop water consumption requirements were calculated based on the soil moisture content of the root zone in the field. The water consumption of winter wheat at each growth stage was calculated based on the principle of water balance, using the following formula:

(2) $$ET=W_{0}-W_{t}+P+I+K-S$$

where *ET* is the water consumption of winter wheat at the defined growth stage (mm), *W*_0_ and *W_t_* are the soil water storage of the target moist soil layer at the beginning of the growth stage and irrigation stage (mm), *P* is the rainfall during the growth stage (mm), *I* is the mean irrigation water amount during the growth stage (mm), *K* is capillary rise during the growth stage (mm), and *S* is drainage below the root zone during the growth stage (mm). The groundwater level at the test site was below 10 m, so the underground water supply was disregarded. In addition, as the depth of the plots was more than 70 cm, there was no disturbance of soil moisture content, so severe leakage could be disregarded (i.e., *S* = 0).

#### 2.3.4. Water-Use Efficiency

WUE was evaluated using the following formula:

(3) WUE=Y/ET

where *WUE* is crop water productivity (kg/ha/mm), *Y* is the yield of winter wheat under nitrogen fertilizer application (t/ha), and *ET* is cropping water consumption (mm).

#### 2.3.5. Nitrogen-Use Efficiency

The partial productivity of nitrogen fertilizer was used to evaluate the nitrogen-use efficiency of winter wheat, using the following formula:

(4) PFPN=Y/F

Here, *PFPN* is the partial productivity of nitrogen fertilizer, *Y* is the yield of winter wheat under nitrogen fertilizer application (t/ha), and *F* is the amount of pure nitrogen applied (kg/ha). *PFPN* refers to the grain yield of crops produced under the specified amount of nitrogen fertilizer.

#### 2.3.6. Harvest Index

*HI* refers to the ratio of economic yield to biomass of a crop, calculated using the following formula:

(5) HI=Y/DM

where *Y* is the yield of winter wheat under nitrogen fertilizer application (t/ha), *HI* is the harvest index, and *DM* is the dry mass of crops at maturity (t/ha).

#### 2.3.7. Plant Height, Dry Matter, and Yield

Three winter wheat plants displaying consistent growth were chosen from the first and second rows within the buffer rows of each experimental plot, resulting in a total of nine recorded observation samples for each treatment, which were then marked using rubber bands. Measurements were taken for the height from the ground to the leaf tip before tillering and from the ground to the spike tip (excluding wheat) after tillering. The average *H* was calculated for each plot.

The drying method was used to determine the dry mass of the stems, leaves, and spikes and the accumulation of *DM* at maturity. After being dried at 105 °C for 30 min to stop respiratory consumption, the samples were further dried at 80 °C until a constant mass was achieved and then weighed and recorded.

Wheat yield measurements were taken from a 3 m^2^ area, with three replicates for each treatment. Within each plot, a random 1 m^2^ sample was selected for harvest and threshing. The effective panicles (*EP*), kernels per spike (*KPS*), and thousand-kernel weight (*TKW*) were assessed using an indoor seed-testing method.

#### 2.3.8. Economic Benefits

The principle of market value was adopted to calculate the economic benefits:

(6) NI=Y×P−Production costs

where *NI* is the economic benefit of winter wheat (CNY), *Y* is the yield of winter wheat, *P* is the unit price of winter wheat (CNY 2.4/kg), and the production cost is CNY/ha. Production cost = human labor cost + production material cost (pesticide cost + fertilizer cost + irrigation cost + seeds cost). Seed cost was CNY 1350/ha, pesticide cost was CNY 300/ha, urea fertilizer cost was CNY 5/kg, potassium fertilizer coat was CNY 4.8/kg, and phosphorus ammonia cost was CNY 6.5/kg at the time of sowing; furthermore, the irrigation water price was 0.2 yuan/m^3^.

### 2.4. Statistical Analysis

Data were sorted and analyzed using Excel, and all processes were set to be repeated 3 times. MANOVA and significance tests were conducted using SPSS26.0 software, and the daily variation in photosynthesis was plotted using Origin 2021. The yield, irrigation, and nitrogen application for winter wheat were analyzed and predicted by using MATLAB R2022.

## 3. Results

### 3.1. Effects of Water–Nitrogen Coupling on Growth Indicators

#### 3.1.1. Plant Height

**Figure 4:**
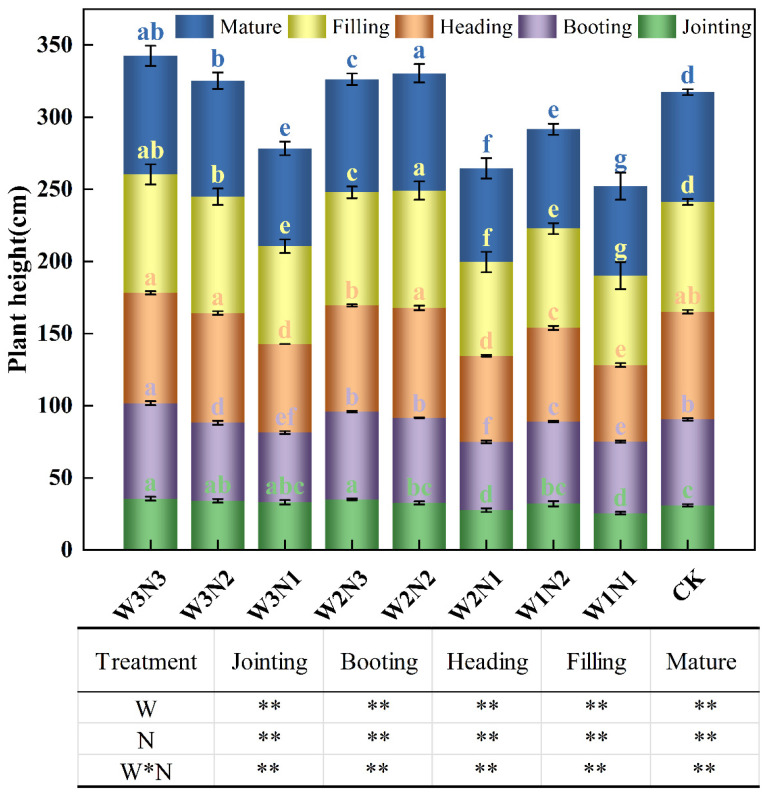
Effects of water and nitrogen coupling on winter wheat plant height (*H*) (cm). Notes: W represents irrigation treatment, N represents nitrogen application treatment, and W*N represents the interaction between water and nitrogen. Different lowercase letters indicate significant differences between different treatments at the 0.05 level (*p* < 0.05). ** indicates that the influence of irrigation, nitrogen application, and the interaction between irrigation and nitrogen in each growth stage is significantly different at 0.01 level (*p* < 0.01).

Plant height (*H*) is a critical indicator of the growth and development of crops in longitudinal space. The effect of water–nitrogen coupling on *H* during the growth period of winter wheat is shown in Figure 4. The results demonstrate that irrigation, nitrogen application, and the interaction between water and nitrogen application have highly significant effects on the *H* of winter wheat throughout the entire growth period (*p* < 0.01). At the jointing stage, the *H* for W1N1 was the lowest, amounting to 25.48 cm, and significantly differed from that for the other treatments (except W2N1) (*p* < 0.05). Under the same nitrogen application rate, the *H* under W1 was lower than that for W2 and W3, indicating that 80% *ET_C_* at the seedling growth period could inhibit the normal development of winter wheat. At the booting and heading stages, there was no significant difference between the W3 and W2 treatments, but there was a significant difference between the W1 and W3 treatments. At the filling stage, the effect of W3 was significantly different compared to that of W2, W1, and CK. *H* tended to be stable during the grain-filling and mature stages. In the mature stage, with increased nitrogen application and consistent irrigation, the *H* of winter wheat increased under W1 and W3, showing an initial rise followed by a declining trend under W2. The various trends in winter wheat *H* at different growth stages were not consistent with different thresholds of nitrogen fertilizer application under different irrigation gradients. Under W3N3, *H* was 82.16 cm, and it was 81.35 cm under W2N2. There was no significant difference between them (*p* > 0.05), but they represented increases of 4.75% and 3.49%, respectively, compared with CK, constituting a significant difference (*p* < 0.05). Compared with local water and nitrogen applications, the *H* of winter wheat increased significantly under experimental conditions. However, there was no significant difference in the *H* of winter wheat under W2N2 compared to W3N3, which corresponded to 20% less *ET_C_* irrigation water and 25% less nitrogen.

#### 3.1.2. Accumulation and Allocation of Dry Matter

##### Dry Matter Accumulation Changes

**Figure 5:**
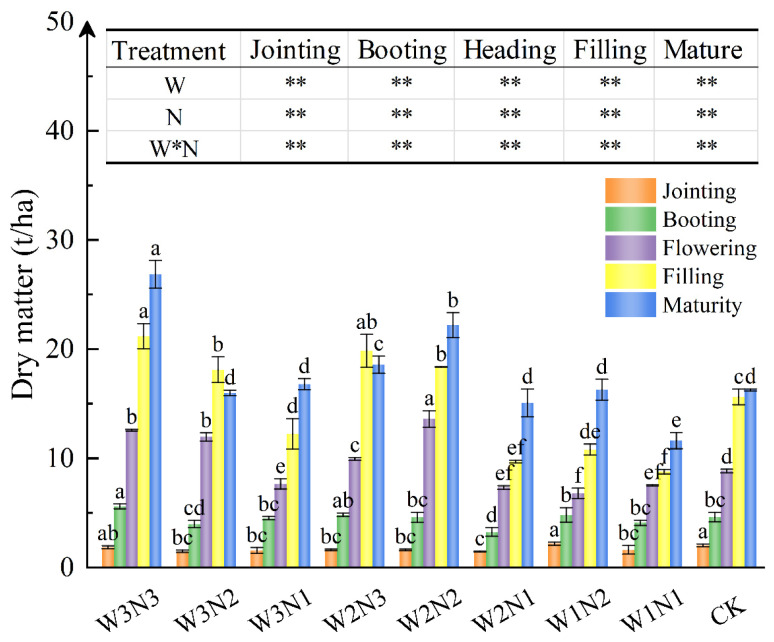
Dry matter (*DM*) accumulation changes during the whole growth period. Different lowercase letters indicate significant differences between different treatments at the 0.05 level (*p* < 0.05). ** indicates that the influence of irrigation, nitrogen application, and the interaction between irrigation and nitrogen in each stage is significantly different at the 0.01 level (*p* < 0.01).

The accumulation trends of total dry matter (*DM*) in winter wheat under different water and nitrogen treatments were similar (Figure 5). Growth was slow in the early stages and then increased rapidly after heading and flowering, with the greatest growth rate occurring during the filling stage, followed by a gradual slowdown. Among the treatments, the total accumulation of *DM* in treatments W3N3 and W2N2 was relatively high, amounting to 26.84 t/ha and 22.19 t/ha, respectively, representing increases of 65.07% and 36.45% compared to the control (CK), indicating they have superior water–nitrogen combinations capable of achieving higher *DM* accumulation. The level of water and fertilizer input in the field directly affects the physiological characteristics of crops, subsequently influencing the accumulation of *DM*, which serves as the material basis for the final yield of crops. Throughout the growth cycle of winter wheat, the accumulation of *DM* continuously increases as the growth stages progress, reaching its peak at the mature stage. In comparisons between treatments, from heading and flowering to maturity, treatment W3N3 consistently outperformed W3N2, and W2N2 consistently outperformed W2N3; treatment W1N2 was only lower than W1N1 during heading and flowering, while it was higher at other times, with significant differences (*p* < 0.05). In summary, in the various irrigation treatments, the application of nitrogen fertilizer can significantly increase the accumulation of DM in winter wheat, but excessive application may not necessarily further increase *DM* accumulation. Multivariate analysis of variance showed that irrigation, nitrogen application, and the interaction of irrigation and nitrogen application had significant effects on the dry matter accumulation of winter wheat (*p* < 0.01), so ensuring water and nitrogen supply was of great significance for the dry matter accumulation of winter wheat.

##### Dry Matter Distribution at Maturity

*DM* accumulation is an important indicator of yield. The cumulative distribution of DM in winter wheat under the different treatments is shown in Table 4. Multivariate analysis of variance revealed that irrigation and the interaction between water and nitrogen had highly significant effects on DM accumulation, as well as on the stems, leaves, and spikes of winter wheat (*p* < 0.01). Conversely, the nitrogen application treatment did not significantly impact DM accumulation in the stems (*p* > 0.05), but it did have significant effects on the leaves and spikes (*p* < 0.05). Moreover, nitrogen application had a significant effect on DM (*p* < 0.01).

The relative DM distribution at the mature stage was as follows: spike > stem > leaf. The maximum amount of DM for stems, leaves, and spikes was generated under W3N3, followed by W2N2. Compared to CK treatment, the stems, leaves and spike of winter wheat under W3N3 treatment increased by 49.54%, 23.01%, and 86.08%, respectively, and those under W2N2 treatment increased by 8.07%, 0%, and 64.21%, respectively; these differences were significant at *p* < 0.05. The total DM under W3N3 (26.84 t/ha) was 65.06% higher than that under CK, followed by W2N2 (22.19 t/ha), for which the total DM was 36.47% higher, and the differences between treatments were significant (*p* < 0.05). Under the same nitrogen application rate, spike DM initially increased and then decreased from W1 to W3. With the same level of irrigation, DM and the proportion of panicles from W1 and W3 increased with nitrogen application. Increasing nitrogen application was the primary means of promoting panicle development under conditions of water scarcity. Under W2, the DM ratio of the spikes could reach more than 60%; the W2N2 spike mass ratio was the highest under all treatments, amounting to 63.28%, which is 64.13% higher than that under CK, and the difference is significant (*p* < 0.05). However, while spike DM increased significantly from N1 to N2, it decreased with N3. There was no significant difference in spike DM between W2N1 and W2N3 (*p* > 0.05). Under N2, the stalk and leaf ratios gradually increased with the increase in the irrigation level, but spike DM initially increased and then decreased; under W3N2, overall DM decreased by 33.69% compared to that for W2N2, and the difference was significant (*p* < 0.05). The combination of water and nitrogen under W2N2 was the most beneficial for grain DM accumulation at the mature stage of winter wheat, which laid the foundation for a high yield.

### 3.2. Effects of Water–Nitrogen Coupling on Physiological Indices

#### 3.2.1. Diurnal Variation in Net Photosynthetic Rate

**Figure 6:**
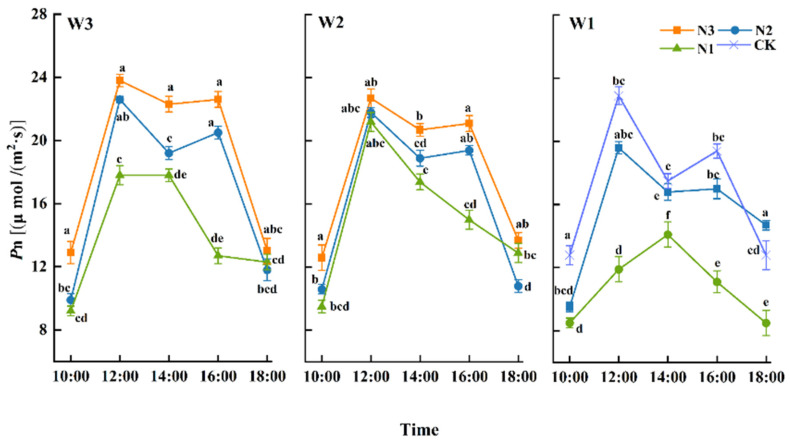
Diurnal variation of net photosynthetic rate (*Pn*) of winter wheat during the filling stage. Different lowercase letters indicate significant differences at the *p* < 0.05 level.

*Pn* is a crucial index reflecting the photosynthetic process of crops. Figure 6 shows the trend of diurnal variation in *Pn* in winter wheat during the grain-filling stage.

At the same irrigation level, winter wheat’s *Pn* exhibited a bimodal trend at nitrogen levels N3 and N2, with peaks at 12:00 and 16:00. A photosynthetic midday depression occurred at around 14:00, and the *Pn* peak at 12:00 was higher than that at 16:00. Under W1, there was often only a single peak. With the same level of irrigation, the trend of *Pn* throughout the day between treatments showed that: W3N3 > W3N2 > W3N1, W2N3 > W2N2 > W2N1, and W1N2 > W1N1. Overall, the *Pn* for the whole day increased with increasing nitrogen application. At the 12:00 peak, the W3N3 and W3N2 levels were 33.71% and 26.97% higher than W3N1, respectively, and the levels of W2N3 and W2N2 were 40.67% and 29.33% higher than W2N1, respectively. At the 16:00 peak, the maximum *Pn* under W3N3 represented a 16.49% increase compared to CK, and the difference was significant (*p* > 0.05). Throughout the day, *Pn* showed a regular positive increase with an increase in nitrogen application, and the effect of N3 on *Pn* was significant. W1N2 and W1N1 each displayed a single peak at 12:00 and 14:00, respectively, with W1N2 representing a 39.01% increase compared to W1N1, indicating that the peak *Pn* value under a low water gradient was consistent with that of a medium–high water gradient. Low fertilizer treatment delayed the peak of *Pn* diurnal variation in winter wheat.

#### 3.2.2. Transpiration Rate (*Tr*)

**Figure 7:**
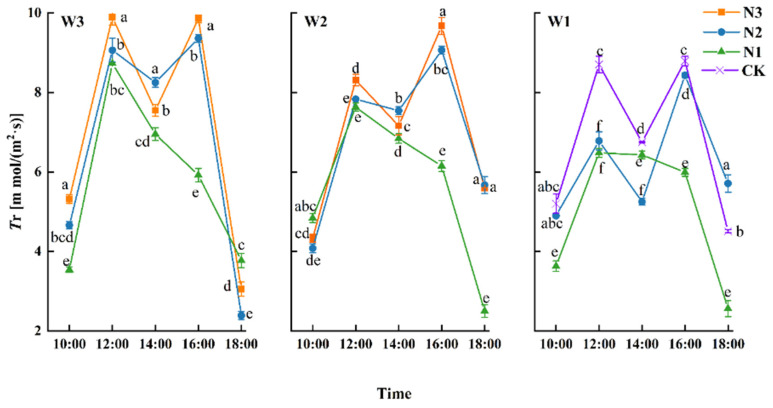
Diurnal variations in transpiration rate (*Tr*) during the filling stage of winter wheat. Different lowercase letters indicate significant differences at the *p* < 0.05 level.

*Tr* reflects the capacity of a crop to metabolize water per unit leaf area during a specified period. Figure 7 shows the diurnal variation of winter wheat *Tr* during grain filling. The diurnal variation in *Tr* was consistent with that of *Pn* in the filling stage for all treatments and showed a double peak from N2 to N3. The photosynthetic midday depression phenomenon co-occurred with *Pn* at 14:00 but showed a single peak under N1. For the whole day, the trend was W3N1 > W2N1 > W1N1. The peak value under W3N1 and W2N1 occurred at 12:00. In contrast, the peak value under W1N1 occurred at 14:00, indicating that W1N1 inhibited *Tr* and delayed the peak of winter wheat daily variation. At 14:00, W2N2 > W2N3 and W3N2 > W3N3, indicating that N3 could force winter wheat leaves to reduce *Tr* at 12:00 to avoid further water loss.

The first peak under differing irrigation levels occurred at 12:00, with W3N3 > W3N2 > W3N1 and W2N3 > W2N2 > W2N1. At the same level of irrigation, the morning peak of *Tr* increased with an increase in the amount of nitrogen fertilizer applied. At 16:00, there was a second peak under W2 and W3, consistent with that at 12:00. However, in contrast to W3, the afternoon peak under W2 was higher than the morning peak. The *Tr* peaks for W2N3 and W2N2 in the afternoon represented increases of 16.37% and 15.71%, respectively, compared with those in the morning, indicating that with the increase in temperature at 12:00, the transpiration rate under W2 was more intense, and a higher fertilizer level could achieve a higher peak. Compared with *Tr* at 14:00, the *Tr* of W3N3 and W3N2 at 16:00 had increased by 30.46% and 13.45%, respectively, and the recovery rate of treatment W3N3 was greater, indicating that increasing nitrogen fertilizer application under a high-water gradient was conducive to maintaining a higher *Tr* for winter wheat during the afternoon.

#### 3.2.3. Leaf Water-Use Efficiency (*L_WUE_*)

**Figure 8:**
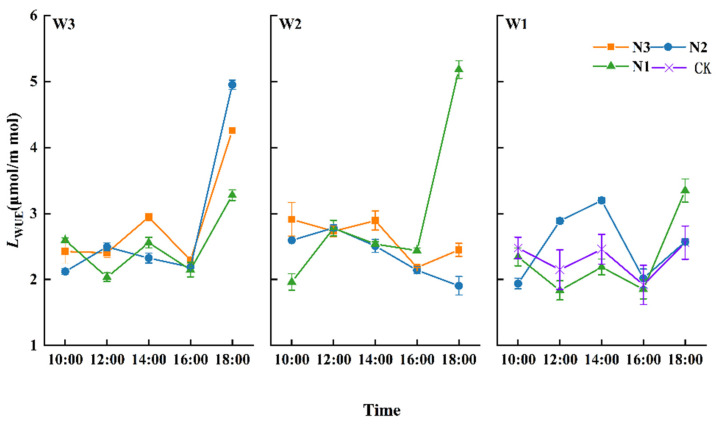
Effects of water and nitrogen coupling on leaf water-use efficiency (*L*_WUE_) of winter wheat.

Leaf water-use efficiency (*L_WUE_*) is a comprehensive physiological and ecological index for measuring crop growth. The diurnal variation in *L_WUE_* in the filling stage for winter wheat is shown in Figure 8.

The *L_WUE_* initially increased and then decreased, before increasing again at 18:00. At 14:00, *L_WUE_* under each water and nitrogen treatment successively reached the first peak of the day as a result of the intense light and high temperatures in southern Xinjiang. Higher temperatures cause stomatal closure in winter wheat, a response initiated to avoid excessive water evaporation and plant wilting.

From 14:00 to 16:00, *L_WUE_* showed a downward trend. This is because *Tr* rises when the temperature drops in the afternoon; transpiration takes away heat, causing leaf temperatures to drop. Although the *Pn* rate of winter wheat showed a second peak at 16:00, the high temperatures increased *Tr*, and the leaf *L_WUE_* decreased by varying degrees. At 18:00, the *L_WUE_* under each water and nitrogen treatment rose successively, caused by the lower temperatures at dusk and the rapid decline in *Tr*. Upon comparing the *L_WUE_* of winter wheat at 14:00, we observed that W1N2 > W3N3 > W2N3 > CK; the *L_WUE_* of winter wheat under N1 was significantly lower than that of CK. Compared with CK, the *L_WUE_* under W3N3 and W2N3 displayed increases of 13.93% and 11.51%; the improvement induced by the higher water treatment was the most significant.

#### 3.2.4. *SPAD*

Chlorophyll is a primary pigment in the thylakoid membrane of crops, and it is responsible for light absorption during photosynthesis. With water and nitrogen coupling, the *SPAD* of winter wheat only showed a single peak throughout the whole growth period (Table 5); at the same time, throughout the entire growth period, irrigation, nitrogen application, and the interaction between water and nitrogen exhibited highly significant effects on *SPAD* values (*p* < 0.01). Overall, *SPAD* values began to decline slowly after reaching a peak during the heading stage. *SPAD* values under W3N3 and W2N2 were relatively high, being 58.44 and 57.90, respectively, 27.18% and 26.01% higher than W1N1 (45.95). Compared with CK, W3N3 and W2N2 increased *SPAD* by 9.03% and 8.02%, respectively, and the differences were significant (*p* < 0.05).

Under the same level of irrigation, *SPAD* under W1 increased when increasing the amount of nitrogen applied. Under W2, the *SPAD* values from the jointing stage to the heading stage ranked as follows: W2N2 > W2N3 > W2N1. However, from the filling stage to the mature stage, the trend was W2N2 > W2N1 > W2N3, and the difference between treatments was significant (*p* < 0.05). Under W3, the changes in *SPAD* at the jointing and booting stages for all treatments were consistent with those for W2, but the following order, W3N3 > W3N2 > W3N1, was observed from the heading stages to the mature stage, and the differences between treatments were significant (*p* < 0.05). With an increase in nitrogen fertilizer from the heading stage to the filling stage, *SPAD* decreased by 10.51%, 19.17%, and 44.35%, respectively. Under W2, increasing nitrogen fertilization at the early growth stage could promote *SPAD*. In contrast, N2 provided the most appropriate nitrogen fertilizer rate at the late growth stage. Increasing the amount of nitrogen fertilizer applied under W3 could increase *SPAD* and delay leaf senescence.

Under N1 and N2, there was no significant difference in *SPAD* for W1, W2, and W3 at the jointing and booting stages. However, the ranking was always W2 > W3 > W1 at the late growth stage, and the difference was significant (*p* < 0.05). Under N3, for the whole growth period, the ranking with respect to *SPAD* was W3N3>W2N3, and the difference was significant (*p* < 0.05). Excessive irrigation during the early growth stage of winter wheat had little effect on improving *SPAD*. In contrast, W2 at the late growing stage was more conducive to higher *SPAD* levels.

### 3.3. Effects of Water–Nitrogen Coupling on Yield and Water- and Nitrogen-Use Efficiency

#### 3.3.1. Water-Use Efficiency

*WUE* refers to the quality of DM produced by a crop per unit mass of water consumption. It directly reflects the energy conversion efficiency in the process of plant production and is an index for analyzing the relationship between crop yield and water consumption. Table 6 shows the effect of water and nitrogen coupling on the *WUE* of winter wheat. It is evident that irrigation treatment, nitrogen application treatment, and the interaction between water and nitrogen all have highly significant effects on winter wheat *WUE* (*p* < 0.01).

Under the same levels of nitrogen fertilizer, W2N1 > W1N1 > W3N1, W2N2 > W3N2 > W1N2, and W3N3 > W2N3. Under N1 and N2, *WUE* initially increased and then decreased with increasing irrigation. The effect of *WUE* under the W3N3 treatment was 16.48% greater than that of W2N3, indicating that W3 could continue to improve *WUE*. Under the same water gradient, *WUE* increased with an increase in nitrogen fertilizer application from N1 to N2. With the increase to N3, the ranking for *WUE* was W2N2 > W2N3 > W2N1, but W3N3 > W3N2 > W3N1. A combination of N2 and W2 therefore appeared to be the most beneficial to winter wheat *WUE*. While the *WUE* of winter wheat could be further improved under W3, there was no significant difference between W3N3 and W2N2 (*p* > 0.05). There were increases of 86.98% and 86.20%, respectively, compared to CK, indicating that reducing the irrigation amount by 20% and nitrogen application by 25% did not significantly reduce the *WUE* of winter wheat. W2N2 was therefore the most beneficial for obtaining a larger *WUE*.

#### 3.3.2. Nitrogen-Use Efficiency and Harvest Index

Enhancing crop *PFPN* and *HI* can lead to reduced production costs and increased overall efficiency. Table 6 presents the *PFPN* and *HI* values for winter wheat under water and nitrogen coupling, demonstrating that irrigation treatment, nitrogen application treatment, and the interaction between water and nitrogen all have highly significant effects on winter wheat’ *PFPN* and *HI* (*p* < 0.01). The maximum *PFPN* obtained under W2N2 was 60.13, which is 35.25% higher than that of CK, and the difference was significant (*p* < 0.05). In addition, under N2, from W1 to W3, *PFPN* initially increased and then decreased with an amount of increasing irrigation, indicating that W2 was more conducive to improving winter wheat *PFPN*.

The *HI* of winter wheat under different water–nitrogen coupling treatments was significantly different (*p* < 0.05). Under the same level of irrigation, the *HI* of winter wheat initially increased and then decreased with an increasing amount of nitrogen applied. The *HI* values of W2N2 were 7.69% and 21.74% higher than those of W2N3 and W2N1, respectively, and this difference was significant (*p* < 0.05). These values for W3N2 were 43.14% and 65.91% higher than those for W3N3 and W3N1, respectively, and, again, the difference was significant (*p* < 0.05), indicating that N2 was more conducive to a higher *HI*. Under the same levels of nitrogen application, *HI* decreased with an increase in irrigation under N3, initially increased and then decreased under N2, and decreased under N1. The highest *HI* was achieved with W3N2 (0.73), and the lowest was achieved with W3N1 (0.44), amounting to a difference of 67.31%. There was no significant difference between CK, W1N1, and W2N2 (*p* > 0.05). Increasing nitrogen fertilizer application positively promoted the *HI* of winter wheat. At the same time, however, excessive irrigation may not be beneficial to improving *HI*.

#### 3.3.3. Yield and Composition

Winter wheat yield and composition are regulated by irrigation and nitrogen. As shown in Table 6, irrigation treatment, nitrogen application treatment, and their interaction had extremely significant effects on yield and *KPS* (*p* < 0.01). Different measures showed varying effects on the *TKW*, with irrigation treatment having a significant effect (*p* < 0.05). Increasing the amount of irrigation water applied could promote more water absorption by winter wheat, thereby stimulating grain growth and weight gain. However, the nitrogen application, and water–nitrogen interaction had a significant effect on the *TKW* (*p* < 0.01). Furthermore, the interaction between irrigation and nitrogen application had no significant effect on the effective panicle number (*p* > 0.05). However, irrigation treatment and nitrogen application each had a significant effect on the effective panicle number (*p* < 0.01).

The effect of water and nitrogen coupling on the final yield of winter wheat corresponded to the following order: W3N3 > W2N2 > W3N2 > W2N2 > CK > W1N2 > W3N1 > W2N1 > W1N1. The most significant yield was under W3N3 (13.599 t/ha), followed by W2N2 (12.447 t/ha). The yields for W3N3 and W2N2 were 47.76% and 35.24% higher than the yield for CK, respectively, and the difference was significant (*p* < 0.05). The minimum yield of 6.571 t/ha was obtained under W1N1, and the difference in yield between W3N3 and W1N1 was as high as 106.94%. The *EP*, *KPS*, 1000-grain mass, and winter wheat yield under different irrigation levels and nitrogen applications increased with an increase in nitrogen amount under W3 but initially increased and then decreased under W2. Under N3, the yield increased with increasing levels of irrigation. Under N2, the yield increased from W1 to W2 and decreased from W2 to W3. In addition, the yield of each treatment under N1 was lower than that of CK, and the corresponding difference was significant (*p* < 0.05), indicating that the nitrogen application rate of N1 was extremely unfavorable for achieving a high yield of winter wheat.

**Figure 9:**
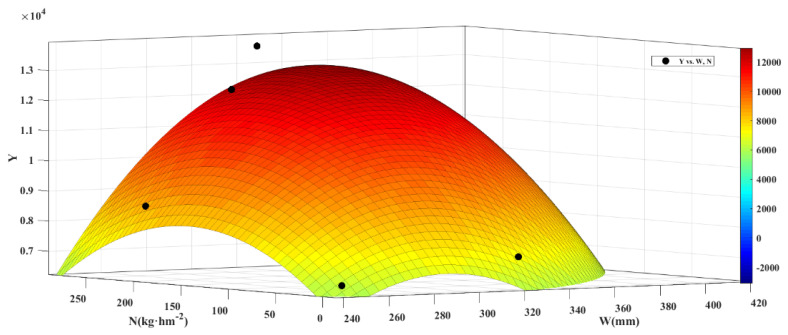
Regression analysis of winter wheat output, irrigation level (*W*), and nitrogen application (*N*).

Figure 9 depicts a three-dimensional graph illustrating the relationship between irrigation level, nitrogen application, and yield. It demonstrates that winter wheat yield initially increases and then decreases under combined water and nitrogen conditions. Moreover, nitrogen application seems to have a critical threshold for enhancing winter wheat yield. At the same irrigation level, increasing nitrogen application initially led to an increase in yield, followed by a decrease. Likewise, increasing irrigation led to an initial rise followed by a decline in yield at the same nitrogen application level. Irrigation, nitrogen application, and their interaction significantly influenced winter wheat yield. The analysis presented in Figure 9 reveals that the highest winter wheat yield, 12.944 t/ha, occurred when the amount of irrigation water was 3420.1 cubic m/ha and the nitrogen application rate reached 251.92 kg/ha.

### 3.4. Effects of Water–Nitrogen Coupling on Economic Efficiency

The final value of winter wheat yield considered was the actual economic benefit. As shown in Table 7, there were significant differences in the economic value, production costs, and final economic benefit of winter wheat under varying water-and-nitrogen-coupling conditions. Under the drip irrigation conditions established in this study, the irrigation costs at the W1, W2, and W3 irrigation levels (Table 2) were CNY 489.9, 565.8, and 646.2, respectively. In comparison, the water cost under CK irrigation was CNY 825, representing increases of 21.67%, 45.81%, and 68.40%, respectively, compared to the aforementioned three irrigation levels. The values under W3N3, W2N2, W2N3, and W3N2 were relatively high, and there were no significant differences between them (*p* > 0.05). The economic value under W3N3 and W2N2 was 47.77% and 35.25%, higher than that for CK, respectively, and the increase in benefit was significant. The economic value for N1 nitrogen application level was lower than CNY 20,000/ha, and there was no significant difference between the treatments (*p* > 0.05). While the levels of output and economic benefit were consistent per treatment, the differences between treatments were significant (*p* < 0.05). The economic benefit of W3N3 and W2N2 was 21,819.9 and 19,885.5 CNY/ha, respectively, 96.74% and 79.30% higher than that for CK (CNY 11,090.7/ha), and this increase is highly significant. The economic benefit under W3N3 was 9.73% higher than that for W2N2. In comparison, the cost of production materials for W3N3 was 13.31% higher than that for W2N2, and the production cost and economic benefit ratios under W3N3 and W2N2 were 0.32 and 0.31, respectively, with no significant differences (*p* > 0.05). W2N2 therefore provided the best economic benefits.

### 3.5. Correlation Analysis between All Indicators

**Figure 10:**
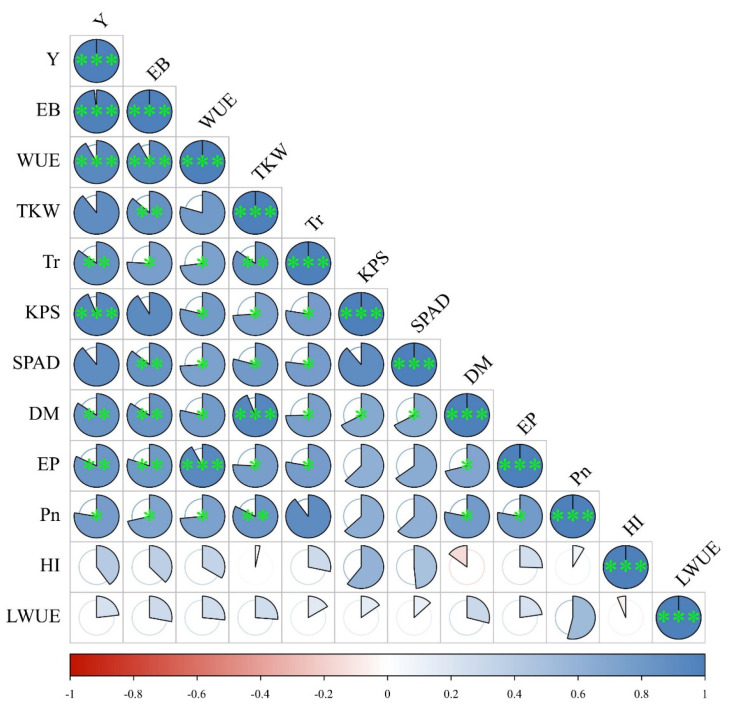
Correlation analysis for all indicators. * indicates a significant correlation at *p* < 0.05, ** indicates a significant correlation at the 0.01 level, *** indicates a significant association at the 0.001 level.

Using the Pearson correlation coefficient, we examined the relationships between 13 indicators, namely, *H*, *DM*, *EP*, *KPS*, *TKW*, *SPAD*, *L_WUE_*, *Pn*, *Tr*, *EB*, *WUE*, *HI*, and *Y*, in order to explore their correlations. Figure 10 illustrates the correlation between growth indicators, physiological indicators, yield and its components, and calculated indicators under the synergistic regulation of water and nitrogen. Through correlation analysis, it was found that winter wheat yield (*Y*) exhibited significantly positive correlations with *H*, *DM*, *EP*, *KPS*, *TKW*, *SPAD*, *Pn*, *Tr*, *EB*, and *WUE* (*p* < 0.01). Among growth and physiological indicators, the strongest correlation with yield was observed for *KPS*, amounting to 0.938, followed by *H*, with 0.934. This suggests that under the synergistic regulation of water and nitrogen, these indicators have a considerable impact on winter wheat yield, indirectly promoting yield through their regulation. However, no significant correlation between Y and *L_WUE_* or *HI* (*p* > 0.05) was observed. Specifically, the correlation between *Y* and *L_WUE_* only amounted to 0.231, indicating a weak association between winter wheat yield and *L_WUE_* and *HI*, suggesting the influence of other factors on their relationship.

## 4. Discussion

### 4.1. Response of Winter Wheat Growth and Development to Water–Nitrogen Interaction

Water scarcity is a key restriction on the sustainable development of agriculture in arid and semi-arid areas [27]. A suitable supply of water and nitrogen can maintain plant growth and development, increasing the functionality of the leaf stage and leaf area index. Therefore, water and nitrogen levels are integral to the production of grain crops such as winter wheat. According to the principles of “promoting fertilizer with water and transferring water with fertilizer”, water and nitrogen input optimization is a reliable way of utilizing water and nitrogen in semi-arid areas efficiently [17]. Under the current experimental conditions, the *H* of winter wheat showed the same trends across the whole growth period under different water and nitrogen treatments. Nitrogen application was conducive to grain filling and advanced the growth period, while low water treatment seriously inhibited plant growth. The maximum *H* was achieved with 100% *ET_C_* irrigation and 207 kg/ha nitrogen application, resulting in a 3.49% increase compared to CK. W3N3 led to the most significant *H* (82.16 cm), followed by W2N2 (81.35 cm), although there were no significant differences between them (*p* < 0.05). This indicated that a 20% water reduction and 25% nitrogen reduction did not limit the growth of winter wheat. MANOVA results revealed that the combined application of water and nitrogen positively impacted the growth and development of winter wheat. Throughout the growth period of winter wheat, irrigation, nitrogen fertilizer application, and the interaction between water and fertilizer significantly influenced *H* and DM (*p* < 0.01), suggesting that the combined application of water and nitrogen fertilizer may have greater effects than individual applications under specific conditions. These factors may require careful management in agricultural production to optimize crop growth and yield.

DM accumulation is a significant contributor to winter wheat yield. Efficient water and nitrogen management can not only advance the rapid accumulation of DM [28] but also increase it. Wang et al. [29] investigated the impact of nitrogen fertilizer application rates ranging from 0 to 300 kg/ha on the dry matter accumulation, distribution, and yield of wheat in Northern Xinjiang. Their study revealed that as the nitrogen fertilizer application rate increased, dry matter accumulation both aboveground and in spikes exhibited an initial increase followed by a decrease. Consequently, it is recommended to decrease the nitrogen fertilizer application amount under drip irrigation to facilitate the migration of dry matter to the spikes, thereby achieving the production objective of increasing yield. Similar conclusions are drawn here; the DM proportion in the lower panicle reached more than 60% under W2N2, laying the foundation for a high yield. The cumulative distribution of DM also affects yield formation, and at maturity, the DM distribution was panicle > stem > leaf. Increasing the level of irrigation under N2 to W3 led to a 33.69% decrease in panicle DM. Excessive water application leads to a plant growth downtrend [30].

### 4.2. Physiological Characteristics of Winter Wheat in Arid Regions

Photo compounds are core factors determining the yield of winter wheat, and about 30% of the DM mass of grain comes from the photosynthesis of flag leaves [31]. Photosynthetic characteristics show regular changes throughout the day as temperature and light conditions change. The most vigorous stage of the photosynthetic process occurs between 12:00 and 16:00. Typical C3 plants experience photosynthetic midday depression, during which there is significantly inhibited photosynthate accumulation. Similar to the conclusions presented in [32], a significant decrease was seen with all treatments at 14:00, but overall, the photosynthetic parameters showed an increasing trend, with bimodal peaks.

A lack of water and nitrogen inhibits *SPAD* and the photosynthetic rate of winter wheat [33]. It may be that the water shortage under 80% *ET_C_* irrigation levels led to the stomatal closure of leaves and a reduction in *Pn* and *Tr*, leading to a delay in the daily peak of photosynthesis. Therefore, adding appropriate amounts of water and nitrogen is more conducive to a positive coupling effect and improving photosynthetic parameters [34], thus increasing yield.

At the 16:00 peak, the *Pn* levels were the highest under W3N3, exhibiting levels that were 16.49% higher than those for CK, and the difference was significant (*p* < 0.05). Water and nitrogen significantly improved the photosynthetic parameters, which is similar to the conclusions in [35]. However, there is a threshold for the influence of water and nitrogen [36]. Under the same level of irrigation, *Pn* and *Tr* increased with an increase in nitrogen. At the same time, *L_WUE_* and *SPAD* values did not maintain a positive and consistent relationship with nitrogen application rates at different growth stages. Furthermore, 100% *ET_C_* and 207 kg/ha were more conducive to higher *SPAD* levels and improved yields. Follow-up studies should be carried out at different growth stages and with different water and fertilizer ratios to determine the optimum conditions for photosynthate accumulation and thus improve yields further.

Under conditions of water and nitrogen coupling, the photosynthetic parameters of winter wheat at the filling stage promoted an increased yield and had a positive effect on its composition. This is consistent with the conclusions of [37], who studied the internal mechanisms of photosynthetic parameters and yield, and of [38], who found that photosynthetic performance is particularly critical to yield formation during grain filling.

### 4.3. Optimizing Yield and Production Efficiency through Water and Nitrogen Application

When promoting the growth and development of winter wheat, it is essential to consider the efficiency of water and nitrogen use when applying water and nitrogen coupling to achieve higher yields and benefits [39]. Applying appropriate water and nitrogen application rates is an effective means of regulating yield components and achieving higher yields. However, as already discussed, there is a threshold for the coupling effect. Ref. [40] found that the yield of winter wheat was higher under irrigation amounts of 376.67 and 378.67 (mm), and the current study reached a similar conclusion, with a maximum yield under W3N3 of 13.599 t/ha. However, according to [41], there is no significant response in yield when nitrogen application rates exceed this level, which is consistent with our conclusions. The reason for this may be that different varieties of winter wheat displayed varying yields and showed unpredictable reactions to the application of nitrogen rates. In this study, nitrogen application had a greater impact on the *TKW* compared to irrigation treatment, especially when considering the interaction between water and nitrogen. This implies that the effect of nitrogen application treatment could be influenced by water availability or may vary under different water conditions. Furthermore, soil parameters significantly influence the relationship between nitrogen application and crop yield. Ignoring soil parameters may lead to an inaccurate interpretation of experimental results; therefore, soil factors should be fully considered in similar studies. For example, soil pH value may affect nitrogen availability and absorption rate, while adsorption complexes in soil can influence nitrogen absorption and release processes. Soils with high adsorption capacity may require higher nitrogen application rates to achieve equivalent crop yields. Irrigation and nitrogen application were identified as independent factors simultaneously influencing the number of *EP*. For example, increased irrigation can promote plant growth and flowering, leading to a higher number of *EP*. Likewise, higher nitrogen application rates may provide more nutrients, promoting winter wheat growth and increasing the number of *EP*. 42. Hamani [40] have proposed that a water-and-nitrogen mode of irrigation is beneficial for attaining higher yields, and splitting nitrogen application (50% basal and 50% topdressing) and applying a 45 mm irrigation quota proved best for winter wheat under drip irrigation, considering yield, evapotranspiration, and water-N productivity. This is similar to the results of this experiment: applying the binary quadratic equation for yield and water and nitrogen use, it was determined that the maximum winter wheat yield was 12.944 t/ha under a combination of 342.01 m^3^/ha of irrigation and 251.92 kg/ha of nitrogen.

The benefit of winter wheat planting is affected by the input as well as yield [42], and the input of production materials such as fertilizers and pesticides is the main reason for the high cost of winter wheat planting [43]. With water and nitrogen coupling, the maximum yield of winter wheat in Southern Xinjiang was 13.599 t/ha under W3N3, and it was 12.447 t/ha under W2N2. However, while W3N3 generated 9.85% more revenue than W2N2, the cost was 13.22% greater than for W2N2; the production cost and economic benefit ratio were 0.46 and 0.45, respectively, and there was no significant difference (*p* > 0.05). This indicates that the production cost of water and nitrogen is not directly proportional to the economic benefit.

Compared with W3N3, W2N2 increased *H* by −0.99% (*p* > 0.05), *PFPN* by 22.04% (*p* < 0.05), *WUE* by −0.4% (*p* > 0.05), and *HI* by 9.8% (*p* < 0.05). W2N2 could therefore save 20% water and 25% nitrogen. W2N2 has higher water- and nitrogen-use efficiency and *HI*, which is more in line with the production needs of agricultural growers. By reducing the input of pre-production materials as much as possible while meeting the required yield and income [44], W2N2 is more conducive to achieving the goal of efficient water and nitrogen utilization and conforms to the development direction of ecological agriculture towards a unity of economic, ecological, and social benefits [45]. The harvest index reflects the ability of a crop to be transformed into economic products and plays a crucial role in evaluations of the yield level and cultivation effect of different crop varieties. However, there is a limitation: the yields of grain and straw cannot be distinguished.

Nitrogen topdressing at different growth stages can improve yields [46], and nitrogen topdressing at a later growth stage can effectively alleviate the contradiction between the supply and demand of nitrogen fertilizer and tillers. In this study, nitrogen fertilizer was applied at the greening, jointing, and booting stages. Compared to the yield generated via the local system of nitrogen fertilizer application (CK), the yield of winter wheat was increased by 47.76%, and the economic benefit was 88.54%. Furthermore, drip irrigation generates more pronounced economic benefits and water-saving effects compared to traditional irrigation methods. This is consistent with the conclusions of previous studies. Under the experimental conditions, W3N3 led to the highest yield, followed by W2N2. However, after a comprehensive comparison of *WUE*, *PFPN*, *HI*, and overall economic benefits, our results indicate that the W2N2 treatment provided the optimal combination of water and nitrogen, taking into account yield, production practices, and economics.

## 5. Conclusions

This study targeted enhancing water and nitrogen utilization efficiency in irrigated winter wheat cultivation in Southern Xinjiang, with the ultimate goal of increasing yield and income. Three main conclusions were obtained based on field experiments:

(1) An irrigation range of 2829–3231 m^3^/ha and a nitrogen application rate of 207 kg/ha resulted in a maximum *H* of 82.16 cm, which was 4.75% higher than that of CK. The DM distribution was as follows: panicle > stem > leaf. Under a combination of 2829 m^3^/ha irrigation and 207 kg/ha nitrogen application, the proportion of DM in the spikes reached more than 60%, and the transport of photosynthates to grains was enhanced, leading to a notable increase in winter wheat yield.
(2) An irrigation range of 2829–3231 m^3^/ha and a nitrogen application rate of 207–276 kg/ha were conducive to accumulating photosynthetic products. No nitrogen application seriously inhibited winter wheat’s photosynthetic parameters, showing a delayed single peak in *Pn* and *Tr*. At the heading stage, *SPAD* values reached their peak value, with optimal levels observed with an irrigation level of 2829 m^3^/ha and a nitrogen application level of 207 kg/ha, peaking at 58.44. Meanwhile, *L*_WUE_ was observed to perform better with an irrigation level of 3231 m^3^/ha and 276 kg/ha of nitrogen fertilizer application.
(3) Considering yield, water and nitrogen use efficiency, and economic benefits, the combination of an irrigation level of 2829 m^3^/ha and a nitrogen application level of 207 kg/ha represented the optimal water and nitrogen combination for the studied site in Southern Xinjiang, China, and a maximum yield of 12.447 t/ha was obtained under the conditions of this study.

This research presents a viable solution for enhancing winter wheat production in arid regions. Subsequent agricultural practices and related research may be centered around irrigation levels of 3420.1 m^3^/ha and nitrogen fertilizer application rates of 251.92 kg/ha to achieve heightened yields. Furthermore, the conclusions drawn from this study can serve as practical guidance for local farmers seeking to refine their cultivation methods, offering both theoretical insights and empirical data that can support further research endeavors in this domain, and the conclusions contribute to the broader discourse on sustainable agriculture, providing practical solutions to addressing challenges such as water scarcity and nutrient management worldwide.

## Figures and Tables

**Table 1:** The properties and initial nutrient content of the soil at the study site.

Soil Layer (cm)	Organic Matter (g/kg)	Bulk Density (g/cm^3^)	Available Nitrogen (mg/kg)	Available Phosphorus (mg/kg)	Quick-Acting Potassium (mg/kg)	Salt (mg/g)	pH	Field Capacity (%)	Soil Texture
0–20	8.97 a + 0.29	1.50 a + 0.01	36.64 a + 0.05	13.98 a + 0.43	115.62 a + 0.28	0.505 a + 0.01	8.57 a + 0.02	22.90 b + 0.15	Silty loam
21–40	6.28 b + 0.21	1.50 a + 0.02	29.61 b + 0.05	6.62 b + 0.27	105.74 a + 0.10	0.505 a + 0.03	8.62 b + 0.01	20.78 c + 0.21	Silty loam
41–60	4.71 c + 0.46	1.45 b + 0.01	21.45 c + 0.09	5.72 b + 0.08	101.81 b + 0.53	0.545 b + 0.03	8.69 b + 0.01	24.99 a + 0.19	Loamy sand

Note: Field water-holding rate = mass water content. Different lowercase letters indicate significant differences between different depth at the 0.05 level (*p* < 0.05).

**Table 2:** Water and nitrogen treatment plan for winter wheat.

Treatment	Irrigation Level	Total Irrigation	Nitrogen Application (kg/ha)			
		m^3^/ha	Bottom Fertilizer	Regreening	Jointing	Heading
W3N3	120% *ET_C_*	3231	138.0	55.2	55.2	27.6
W3N2	120% *ET_C_*	3231	138.0	41.4	41.4	20.7
W3N1	120% *ET_C_*	3231	138.0	0	0	0
W2N3	100% *ET_C_*	2829	103.5	55.2	55.2	27.6
W2N2	100% *ET_C_*	2829	103.5	41.4	41.4	20.7
W2N1	100% *ET_C_*	2829	103.5	0	0	0
W1N2	80% *ET_C_*	2449.5	0	41.4	41.4	20.7
W1N1	80% *ET_C_*	2449.5	0	0	0	0
CK	-	4125	103.5	34.5	34.5	34.5

Note: *ET_C_* is the amount of crop water needed.

**Table 3:** *Kc* value of winter wheat at different growth stages.

Growth	Regreening	Jointing	Heading	Filling	Mature
*Kc*	0.85	1.02	1.16	0.74	0.9425

**Table 4:** Effects of coupling of water and nitrogen on the accumulation and distribution of dry matter (*DM*) in winter wheat at maturity.

Treatment		Stem		Leaf		Spike		Total DM
		DM	Ratio/%	DM	Ratio/%	DM	Ratio/%	DM
		(t/ha)		(t/ha)		(t/ha)		(t/ha)
W3N3		8.15 a	30.37%	2.78 a	10.36%	15.91 a	59.27%	26.84 a
W3N2		4.91 b	30.67%	1.78 bc	11.13%	9.31 cd	58.20%	16.00 d
W3N1		5.68 b	33.84%	2.07 abc	12.34%	9.04 cd	53.82%	16.79 d
W2N3		5.67 b	30.50%	2.08 abc	11.19%	10.83 bc	58.31%	18.57 c
W2N2		5.89 b	26.52%	2.26 ab	10.20%	14.04 ab	63.28%	22.19 b
W2N1		4.55 b	30.15%	1.25 c	8.31%	9.28 cd	61.54%	15.07 d
W1N2		4.92 b	30.17%	1.69 bc	10.35%	9.69 cd	59.47%	16.29 d
W1N1		4.02 b	34.61%	1.38 bc	11.88%	6.22 d	53.51%	11.62 e
CK		5.45 b	33.49%	2.26 ab	13.90%	8.55 cd	52.61%	16.26 d
MANOVA	W	**		**		**		**
	N	None		*		*		**
	W*N	**		**		**		**

DM: dry matter (t/ha). Different lowercase letters indicate significant differences at the *p* < 0.05 level. * indicates that the influence of irrigation, nitrogen application, and the interaction between irrigation and nitrogen at the mature stage is significantly different at 0.05 level (*p* < 0.05), ** indicates significant difference at 0.01 level (*p* < 0.01), and None means no significant difference (*p* > 0.05).

**Table 5:** Effects of water and nitrogen coupling on *SPAD* levels of winter wheat.

Treatment		Growth Stage				
		Jointing	Booting	Heading	Filling	Mature
W3N3		51.37 a	53.57 a	58.44 a	52.30 a	12.40 a
W3N2		51.67 a	53.93 a	56.33 b	45.53 b	8.53 bc
W3N1		39.20 d	43.17 d	47.80 e	26.60 d	3.40 e
W2N3		43.60 c	49.97 b	53.90 c	25.40 d	7.43 cd
W2N2		50.83 a	52.37 a	57.90 a	54.93 a	10.17 a
W2N1		43.53 c	45.93 c	46.70 f	35.50 c	8.51 bc
W1N2		48.95 b	51.25 a	51.00 d	28.95 d	7.00 cd
W1N1		44.35 c	46.55 c	45.95 g	25.55 d	4.90 de
CK		48.35 b	51.75 a	53.60 c	53.00 a	7.95 bc
MANOVA	W	**	**	**	**	**
	N	**	**	**	**	**
	W*N	**	**	**	**	**

Different lowercase letters indicate significant differences at the *p* < 0.05 level. ** indicates that the influence of irrigation, nitrogen application, and the interaction between irrigation and nitrogen in each stage is significantly different at 0.01 level (*p* < 0.01).

**Table 6:** Effects of water and nitrogen coupling on winter wheat yield and composition, water- and nitrogen-use efficiency, and harvest index (*HI*).

Treatment		Effective Panicles (*EP*)	Kernels per Spike (*KPS*)	Thousand Kernel Weight (*TKW*)	Yield	*HI*	*WUE*	*PFPN*
		(grain)	(Induvial)	(g)	(t/ha)		kg/(ha·mm)	
W3N3		46.77 b	654 a	52.38 a	13.599 a	0.51 e	39.58 b	49.27 c
W3N2		46.63 b	630 b	46.64 c	11.644 c	0.73 a	33.98 c	56.25 b
W3N1		42.05 cd	448 g	45.34 cd	7.304 f	0.44 g	20.11 f	-
W2N3		47.05 b	524 e	45.99 c	9.614 d	0.52 d	31.25 d	34.83 f
W2N2		49.95 a	592 c	49.52 b	12.447 b	0.56 b	39.42 ab	60.13 a
W2N1		42.53 c	435 h	43.74 d	6.911 fg	0.46 f	21.77 f	-
W1N2		45.75 b	478 f	46.62 c	8.679 e	0.53 c	30.28 d	41.93 de
W1N1		39.7 d	483 f	40.28 e	6.571 g	0.57 b	21.20 e	-
CK		42.08 cd	554 d	46.62 c	9.203 d	0.57 b	21.17 f	44.46 d
MANOVA	W	**	**	*	**	**	**	**
	N	**	**	**	**	**	**	**
	W*N	None	**	**	**	**	**	**

Different lowercase letters in the same column represent significant differences between treatments (*p* < 0.05). * indicates that the influence of irrigation, nitrogen application, and the interaction between irrigation and nitrogen is significantly different at 0.05 level (*p* < 0.05), ** indicates significant difference at 0.01 level (*p* < 0.01), and None means no significant difference.

**Table 7:** Effects of water and nitrogen coupling on economic benefits of winter wheat.

Treatment		Economic Value	Production Costs (CNY/ha)			Economic Benefit
		(CNY/ha)	Labor Cost	Mechanical Cost	Production Materials Cost	(CNY/ha)
W3N3		32,637.6 a	2250 a	1500 a	7067.7 b	21,819.9 a
W3N2		27,945.6 ab	2250 a	1500 a	6317.7 d	17,877.9 c
W3N1		17,529.6 c	2250 a	1500 a	4067.7 g	9711.9 g
W2N3		23,073.6 abc	2250 a	1500 a	6987.3 c	12,336.3 d
W2N2		29,872.8 ab	2250 a	1500 a	6237.3 e	19,885.5 b
W2N1		16,586.4 c	2250 a	1500 a	3987.3 h	8849.1 h
W1N2		20,829.6 bc	2250 a	1500 a	6161.4 f	10,918.2 f
W1N1		15,770.4 c	2250 a	1500 a	3911.4 i	8109.0 i
CK		22,087.2 bc	2250 a	1500 a	7246.5 a	11,090.7 e
MANOVA	W	**	**			**
	N	**	**			**
	W*N	**	**			**

Different lowercase letters indicate significant differences at the *p* < 0.05 level. ** indicates that the influence of irrigation, nitrogen application, and the interaction between irrigation and nitrogen is significantly different at 0.01 level (*p* < 0.01).

## Data Availability

Data are contained within the article.

## References

[1] Yermiyahu U., Ben-Gal A., Keren R., Reid R.J. (2008). Combined Effect of Salinity and Excess Boron on Plant Growth and Yield. Plant Soil.

[2] Mupambwa H.A., Hausiku M.K., Nciizah A.D., Dube E. (2019). The Unique Namib Desert-Coastal Region and Its Opportunities for Climate Smart Agriculture: A Review. Cogent Food Agric..

[3] Ning S., Zhou B., Shi J., Wang Q. (2021). Soil Water/Salt Balance and Water Productivity of Typical Irrigation Schedules for Cotton under Film Mulched Drip Irrigation in Northern Xinjiang. Agric. Water Manag..

[4] Ma L., Zhang X., Lei Q., Liu F. (2021). Effects of Drip Irrigation Nitrogen Coupling on Dry Matter Accumulation and Yield of Summer Maize in Arid Areas of China. Field Crops Res..

[5] Che Z., Wang J., Li J. (2021). Effects of Water Quality, Irrigation Amount and Nitrogen Applied on Soil Salinity and Cotton Production under Mulched Drip Irrigation in Arid Northwest China. Agric. Water Manag..

[6] Qinglin N., Haikuan F., Xinguo Z., Jianqiang Z., Beibei Y., Huizhen L. (2021). Combining UAV Visible Light and Multispectral Vegetation Indices for Estimating SPAD Value of Winter Wheat. Trans. Chin. Soc. Agric. Mach..

[7] Enguwa K.B.P., Horn L.N., Awala S.K. (2024). Comparative Effect of Different Irrigation Levels and Soil Amendments on Cabbage Productivity in Semi-arid Central Namibia. Irrig. Drain..

[8] Hunsaker D.J., French A.N., Waller P.M., Bautista E., Thorp K.R., Bronson K.F., Andrade-Sanchez P. (2015). Comparison of Traditional and ET-Based Irrigation Scheduling of Surface-Irrigated Cotton in the Arid Southwestern USA. Agric. Water Manag..

[9] Zain M., Si Z., Li S., Gao Y., Mehmood F., Rahman S.-U., Mounkaila Hamani A.K., Duan A. (2021). The Coupled Effects of Irrigation Scheduling and Nitrogen Fertilization Mode on Growth, Yield and Water Use Efficiency in Drip-Irrigated Winter Wheat. Sustainability.

[10] Yang P., Hu H., Tian F., Zhang Z., Dai C. (2016). Crop Coefficient for Cotton under Plastic Mulch and Drip Irrigation Based on Eddy Covariance Observation in an Arid Area of Northwestern China. Agric. Water Manag..

[11] Wang F., Xie R., Ming B., Wang K., Hou P., Chen J., Liu G., Zhang G., Xue J., Li S. (2021). Dry Matter Accumulation After Silking Kernel Weight. Are Key Factors Increasing Maize Yield Water Use Efficiency. Agric. Water Manag..

[12] Chenxiao Y., Ming H., Jiahao Q., Junjie F., Haijun L., Yun W. (2021). Effects of Biochar on Hydraulic Characteristics of Aeolian Sandy Soil in Hetian. Agric. Res. Arid. Areas.

[13] Li J., Wang Z., Song Y., Li J., Zhang Y. (2022). Effects of Reducing Nitrogen Application Rate under Different Irrigation Methods on Grain Yield, Water and Nitrogen Utilization in Winter Wheat. Agronomy.

[14] Liu P., Tu M., Song H., Chen D., Sun S., Li J., Xu Z., Liu C., Lin L., Jiang G. (2021). Effects of Rain-Shelter Cultivation on Physiological and Biochemical Indexes of Kiwifruit Leaves and Fruit Quality. Southwest China J. Agric. Sci..

[15] Sun W., Hamani A.K.M., Si Z., Abubakar S.A., Liang Y., Liu K., Gao Y. (2022). Effects of Timing in Irrigation and Fertilization on Soil NO_3_^−^-N Distribution, Grain Yield and Water–Nitrogen Use Efficiency of Drip-Fertigated Winter Wheat in the North China Plain. Water.

[16] Xu J., Lv Y., Liu X., Wei Q., Qi Z., Yang S., Liao L. (2019). A General Non-Rectangular Hyperbola Equation for Photosynthetic Light Response Curve of Rice at Various Leaf Ages. Sci. Rep..

[17] Zhang M., Sun D., Niu Z., Yan J., Zhou X., Kang X. (2020). Effects of Combined Organic/Inorganic Fertilizer Application on Growth, Photosynthetic Characteristics, Yield and Fruit Quality of Actinidia Chinesis Cv ‘Hongyang’. Glob. Ecol. Conserv..

[18] Ren B., Yu W., Liu P., Zhao B., Zhang J. (2023). Responses of Photosynthetic Characteristics and Leaf Senescence in Summer Maize to Simultaneous Stresses of Waterlogging and Shading. Crop J..

[19] Goosheh M., Pazira E., Gholami A., Andarzian B., Panahpour E. (2018). Improving Irrigation Scheduling of Wheat to Increase Water Productivity in Shallow Groundwater Conditions Using Aquacrop. Irrig. Drain..

[20] Kubar M.S., Alshallash K.S., Asghar M.A., Feng M., Raza A., Wang C., Saleem K., Ullah A., Yang W., Kubar K.A. (2022). Improving Winter Wheat Photosynthesis, Nitrogen Use Efficiency, and Yield by Optimizing Nitrogen Fertilization. Life.

[21] Liu K., Shi Y., Yu Z., Zhang Z., Zhang Y. (2023). Improving Photosynthesis and Grain Yield in Wheat through Ridge–Furrow Ratio Optimization. Agronomy.

[22] Tavakoli A.R., Sepaskhah A.R., Hokmabadi H. (2023). Introducing a Stratified Vertical Gravel Tube Subsurface Drip System under Different Irrigation Regimes for Pistachio: Growth, Yield and Water Productivity. Irrig. Drain..

[23] She Y., Li P., Du Z., Qi X., Zhao S., Li T., Guo W. (2022). Nitrogen Fertilization Effects on Soil Nitrate, Water Use, Growth Attributes and Yield of Winter Wheat under Shallow Groundwater Table Condition. Agronomy.

[24] Li Y., Ma L., Wu P., Zhao X., Chen X., Gao X. (2020). Yield, Yield Attributes and Photosynthetic Physiological Characteristics of Dryland Wheat (*Triticum aestivum* L.)/Maize (*Zea mays* L.) Strip Intercropping. Field Crops Res..

[25] Hu J., Zhao X., Gu L., Liu P., Zhao B., Zhang J., Ren B. (2023). The Effects of High Temperature, Drought, and Their Combined Stresses on the Photosynthesis and Senescence of Summer Maize. Agric. Water Manag..

[26] Zhou B., Liang C., Chen X., Ye S., Peng Y., Yang L., Duan M., Wang X. (2022). Magnetically-Treated Brackish Water Affects Soil Water-Salt Distribution and the Growth of Cotton with Film Mulch Drip Irrigation in Xinjiang, China. Agric. Water Manag..

[27] Chen Z., Zhang X., Chen J. (2021). Monitoring Terrestrial Water Storage Changes with the Tongji-Grace2018 Model in the Nine Major River Basins of the Chinese Mainland. Remote Sens..

[28] Lee C.-C., He Z.-W., Luo H.-P. (2024). Spatio-Temporal Characteristics of Land Ecological Security and Analysis of Influencing Factors in Cities of Major Grain-Producing Regions of China. Environ. Impact Assess. Rev..

[29] Wang R., Wang H., Jiang G., Liu J., Yin H., Xie B., Che Z., Jiang F., Zhang T. (2022). Effect of Nitrogen Application on Root and Yield Traits of Chinese Spring Wheat (*Triticum aestivum* L.) under Drip Irrigation. Agronomy.

[30] Qiu S., Ju X., Lu X., Li L., Ingwersen J., Streck T., Christie P., Zhang F. (2012). Improved Nitrogen Management for an Intensive Winter Wheat/Summer Maize Double-cropping System. Soil Sci. Soc Am. J..

[31] Xu J., Zhao H., Wang S., Zheng Y., Mai B., Zhang X. (2023). Assessment of Photosynthesis and Yield Loss of Winter Wheat under Ground-Level Ozone Exposure. Environ. Technol. Innov..

[32] Ma M., Liu Y., Zhang Y., Qin W., Wang Z., Zhang Y., Lu C., Lu Q. (2021). In Situ Measurements of Winter Wheat Diurnal Changes in Photosynthesis and Environmental Factors Reveal New Insight into Photosynthesis Improvement by Super-High-Yield Cultivation. J. Integr. Agric..

[33] Yang X., Yang R., Ye Y., Yuan Z., Wang D., Hua K. (2021). Winter Wheat SPAD Estimation from UAV Hyperspectral Data Using Cluster-Regression Methods. Int. J. Appl. Earth Obs. Geoinf..

[34] Cao J.-L., Wang L., Zeng Q., Liang J., Tang H.-Y., Xie Z.-B., Liu G., Zhu J.-G., Kobayashi K. (2009). Characteristics of Photosynthesis in Wheat Cultivars with Different Sensitivities to Ozone Under O3-Free Air Concentration Enrichment Conditions. Acta Agron. Sin..

[35] Li Y., Gu X., Li Y., Fang H., Chen P. (2023). Ridge-Furrow Mulching Combined with Appropriate Nitrogen Rate for Enhancing Photosynthetic Efficiency, Yield and Water Use Efficiency of Summer Maize in a Semi-Arid Region of China. Agric. Water Manag..

[36] Song K., Lu Y., Dao G., Chen Z., Wu Y., Wang S., Liu J., Hu H.-Y. (2022). Reclaimed Water for Landscape Water Replenishment: Threshold Nitrogen and Phosphorus Concentrations Values for Bloom Control. Algal Res..

[37] Zhang S., Gong J., Xiao C., Yang X., Li X., Zhang Z., Song L., Zhang W., Dong X., Hu Y. (2024). Bupleurum Chinense and Medicago Sativa Sustain Their Growth in Agrophotovoltaic Systems by Regulating Photosynthetic Mechanisms. Renew. Sustain. Energy Rev..

[38] Liu J., Si Z., Li S., Wu L., Zhang Y., Wu X., Cao H., Gao Y., Duan A. (2023). Effects of Water and Nitrogen Rate on Grain-Filling Characteristics under High-Low Seedbed Cultivation in Winter Wheat. J. Integr. Agric..

[39] Han M., Zhang J., Zhang L., Wang Z. (2023). Effect of Biochar Addition on Crop Yield, Water and Nitrogen Use Efficiency: A Meta-Analysis. J. Clean. Prod..

[40] Hamani A.K.M., Abubakar S.A., Si Z., Kama R., Gao Y., Duan A. (2023). Responses of Grain Yield and Water-Nitrogen Dynamic of Drip-Irrigated Winter Wheat (*Triticum aestivum* L.) to Different Nitrogen Fertigation and Water Regimes in the North China Plain. Agric. Water Manag..

[41] Azizah F.N., Purwanto B.H., Tawaraya K., Rachmawati D. (2023). Characterization of Yield and Cumulative Nitrous Oxide Emission of Maize Varieties in Responses to Different Nitrogen Application Rates. Heliyon.

[42] Zhang K., Wang X., Li Y., Zhao J., Yang Y., Zang H., Zeng Z. (2022). Peanut Residue Incorporation Benefits Crop Yield, Nitrogen Yield, and Water Use Efficiency of Summer Peanut—Winter Wheat Systems. Field Crops Res..

[43] Li S., Wang S., Shi J., Tian X., Wu J. (2022). Economic, Energy and Environmental Performance Assessment on Wheat Production under Water-Saving Cultivation Strategies. Energy.

[44] Attipoe S.G., Cao J., Opoku-Kwanowaa Y., Ohene-Sefa F. (2021). Assessing the Impact of Non-Governmental Organization’s Extension Programs on Sustainable Cocoa Production and Household Income in Ghana. J. Integr. Agric..

[45] Feng Y., Zhu A., Liu P., Liu Z. (2022). Coupling and Coordinated Relationship of Water Utilization, Industrial Development and Ecological Welfare in the Yellow River Basin, China. J. Clean. Prod..

[46] Tian Y., Zhao X., Yin B., Yan X. (2023). Delaying Tillering Nitrogen Topdressing until the Midtillering Phase Improves Nitrogen Use Efficiency and Reduces Ammonia Emission via Rice Canopy Recapture. Eur. J. Agron..

